# Case report: An OSAS patient with comorbid RLS/PLMS – a closer look at the causes of PAP intolerance

**DOI:** 10.3389/fneur.2023.1257736

**Published:** 2023-10-11

**Authors:** Christina A. H. Dirks, Cornelius G. Bachmann

**Affiliations:** SomnoDiagnostics, Osnabrück, Germany

**Keywords:** hypoglossal nerve stimulation, tongue pacemaker, obstructive sleep apnea, restless legs syndrome, periodic limb movements in sleep, augmentation, PAP-therapy

## Abstract

Since 2017, hypoglossal nerve stimulation has been included in the S3-guidelines on restorative sleep/sleep disorders as an alternative treatment for patients with obstructive sleep related breathing disorders who cannot tolerate conventional PAP-therapy. Under certain conditions, some of these patients have the option to have a tongue pacemaker implanted during a surgical procedure to regain a restful night’s sleep. However, in some cases it does not solve the problem. In this case report, we present a patient who continued to have restless sleep despite implantation of a hypoglossus nerve stimulator. We provide a closer look at the underlying causes of PAP intolerance and emphasize the importance of a combined pneumological and neurological approach to sleep medicine in sleep-specific therapy evaluation.

## Introduction

Initiating PAP-therapy is frustrating for many patients diagnosed with obstructive sleep apnea syndrome. This therapy represents the gold standard for treatment because it is non-invasive and has almost no known side effects, though many patients experience air leakage due to nocturnal movements, insomnia or anxiety disorder triggered by the feeling of tightness in the fitting of the mask. The appearance of the mask and the presumed negative effect on the patient’s partner also may deter some patients from using the APAP mask regularly.

Since 2017, hypoglossal nerve stimulation has been included in the S3-guidelines on restorative sleep/sleep disorders as an alternative treatment for patients who cannot tolerate conventional PAP-therapy. Up until now, around 40.000 tongue pacemakers have been implanted and their effectiveness has been proven in various scientific studies. Therapy for obstructive sleep apnea that involves stimulating the hypoglossal nerve (tongue pacemaker) is an alternative treatment method for patients who have moderate to severe obstructive sleep apnea syndrome (OSAS), a BMI of <35 kg/m^2^, no other sleep disorders and no other neuromuscular disorders. If these criteria are met, a pacemaker can be implanted with a minor surgical procedure. Tongue pacemaker surgery is performed in a specialized treatment center under general anesthesia, and requires two to three small incisions in the neck and chest. Independently, patient can then switch the tongue pacemaker on in the evening and off again in the morning using a remote control device.

On the other hand, restless legs syndrome (RLS) is a sleep disorder, in which the restless legs sensations cannot be switched off by the push of a button. RLS is characterized by abnormal sensations in the legs or – more rarely – in the arms as well as in the bladder, rectal or genital region. Such sensations appear, at least initially, with a circadian rhythm when the patient is at rest and are associated with an urge to move. In 85% of the patients, RLS is correlated with periodic limb movements during sleep (PLMS) and symptoms decrease due to movement of the affected body parts.

RLS is a common neurological disease with a prevalence of 7%–10% observed in the populations of western industrialized countries (1). The new S2k-guidelines for the treatment of RLS recommend drug treatment with Ropinirole, Rotigotine or Pramipexole at the lowest effective dose to avoid augmentation effects. Due to the high augmentation rates at all doses of Levodopa, especially at doses ≥200 mg, and the potential risk of excessive self-medication, the use of Levodopa as an RLS treatment is not recommended.

Augmentation is defined as a spread of abnormal sensations to previously unaffected parts of the body, a need to increase the dose of the medication or the temporal advancement of the symptoms throughout the day. It has been postulated that augmentation results from compensatory mechanisms of the dopaminergic system which may lower the tolerance of dopaminergic effects, particularly in relation to the circadian system. In most cases, patients who suffer from an augmentation do not attribute this to their medication. Rather, they believe it is an exacerbation of the disease and, consequently, take continuously higher doses of their medication which, in turn, further exacerbates augmentation.

## Report of case

A 44-year-old male patient, 188 cm, 105 kg, BMI = 29.7 kg/m^2^, was admitted to our sleep laboratory for the first time in July 2020 for a check-up of the tongue pacemaker that was implanted in June 2019. Previous findings revealed a severe obstructive sleep apnea syndrome (AHI = 31/h, ODI = 23/h, min SaO2 = 80%, basal saturation = 94%), restless legs syndrome, ex-nicotine abuse (15/d, 30 PY), arterial hypertension, depressive disorder, bruxism and myalgia in the forearms, which has been observed since an accident that occurred in 1998. During the patient’s history interview, the patient reported that he has not adapted well to the tongue pacemaker. Though he has already reached the highest pulse level of the tongue pacemaker, he continued to experience long breathing pauses at night. In addition, the patient reported a persistent daytime sleepiness (ESS = 15 points), unrefreshing sleep at night and discomfort in the legs when resting with an urge to move. A subsequent neurological examination had been made without pathological findings, however, a family history of RLS was reported, though RLS-relevant blood values were normal, in particular, the ferritin value was = 130 μg/L, and an exclusion of the presence of a polyneuropathy by nerve conduction velocity measurement was noted. This patient had already been prescribed Levodopa by his neurologist. It was not unexpected that he had already showed signs of augmentation, such as the need to steadily increase his dosage to maintain the same level of medication effectiveness. He reportedly took Levodopa at a dosage of 500/125 mg throughout the day.

The patient had undertaken several unsuccessful attempts to initiate APAP-therapy. These efforts failed mainly due to air leaks in the mask, the result of his increased movements at night.

### Medication at July 2020

Bisoprolol 10 mg 0-0-2.

Doxepin 100 mg 0-0-1.

Levodopa 100/25 mg 0-0-2 (in the meantime increased to 500/125 mg per day).

Pantoprazol 20 mg 2-0-0.

Before the first night of his cardiorespiratory polysomnography, the sleep laboratory staff reported that the hypoglossal stimulator interacted with some of the technical devices from the sleep laboratory bluetooth system. For this reason, the subsequent recording was carried out without the tongue pacemaker results every night. In the evening before going to bed, the patient was observed to be very restless and the camera showed him getting up several times during the course of night to move his legs and arms even though he took his usual medication of Pantoprazol 40 mg, Bisoprolol 20 mg, Doxepin 100 mg and Levodopa 500/125 mg.

**Figure 1:**
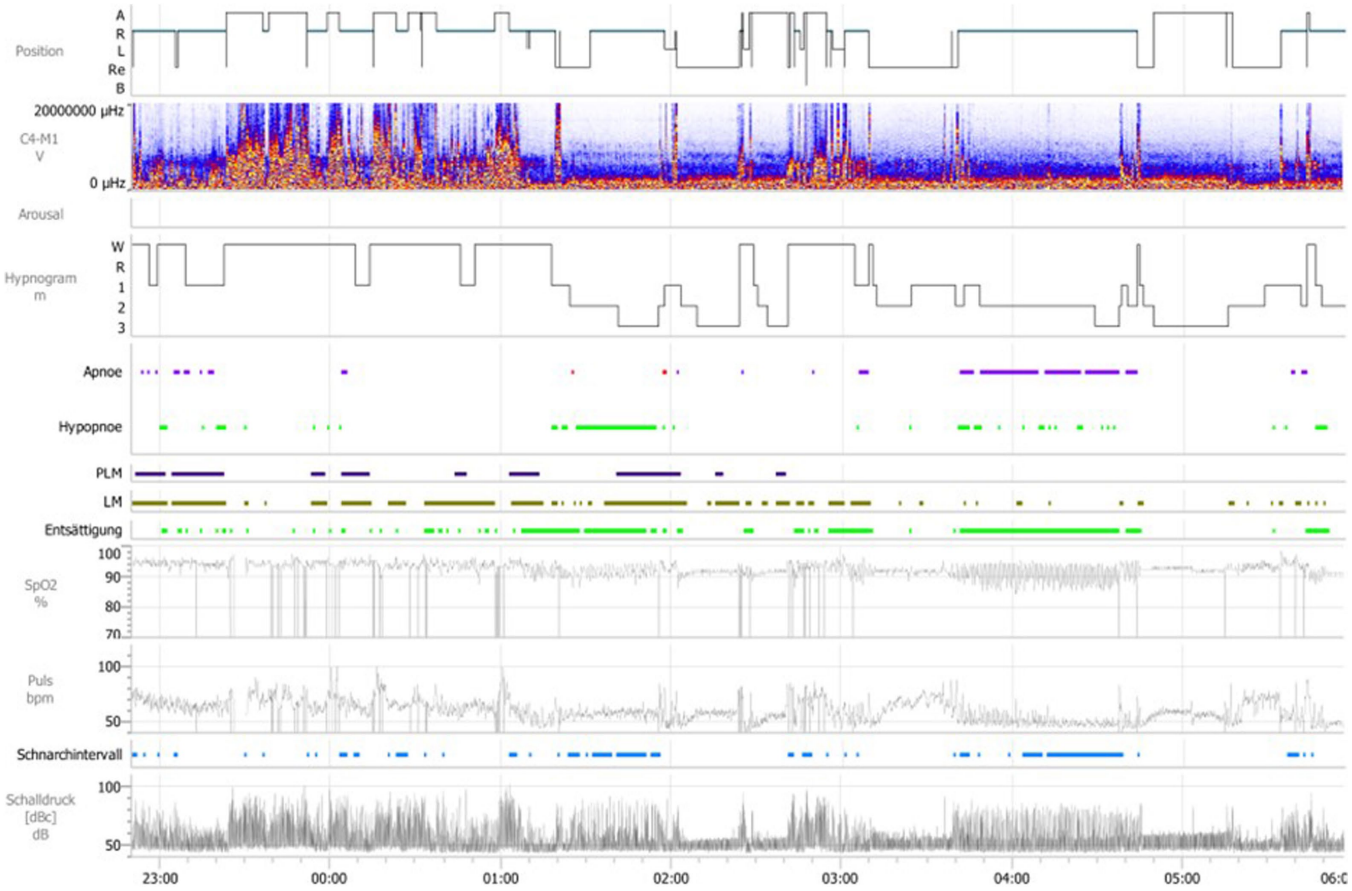
General overview over the first polysomnography, first night. Position A = upright position, Position R = on the back, Position L = left side, Position Re = right side, Position B = on the stomach, μHZ = microhertz, C4-M1 V = Derivation of EEG electrodes C4 to M1, Hypnogramm W = wake, Hypnogramm R = REM-Sleep, Hypnogramm 1 = sleep stage 1. Hypnogramm 2 = sleep stage 2, Hypnogramm 3 = sleep stage 3, PLM = periodic limb movement, LM = limb movement, SpO2% = oxygen saturation in %, Puls bpm = beats per minute, Schnarchintervall = snoring interval, Schalldruck db = sound pressure in decibel.

As expected, the first night of polysomnography (Figure 1) showed the untreated, severe obstructive sleep apnea syndrome and an increased PLM index while taking his RLS-medication of 500/125 mg Levodopa in the evening (for detailed overview over the PSG-parameters see Table 1).

**Table 1:** Overview of the PSG parameters in all four monitoring nights.

PSG-parameter	1. PSG first night (augmentation under 500/125 mg Levodopa)	1. PSG second night (medication changed to Rotigotine 3 mg/24 h, Tilidin 50/4 mg)	2. PSG first night (medication changed to Ropinirol 1 mg and retard 2 mg, Tilidin 50/4 mg, Pregabalin 25-50-75 mg)	2. PSG second night (no medication change + APAP-therapy)
TIB	426,8 min.	407,9 min.	413 min.	421 min.
TST	272,5 min.	384 min.	365,3 min.	405,7 min.
Sleep efficiency	63,9%	94,1%	88,4%	96,4%
REM	0%	0%	6,8%	11,6%
N1	31,7%	1,6%	3,8%	3,5%
N2	42,2%	88,9%	59,5%	48,1%
N3	26,1%	9,5%	29,8%	36,8%
Sleep onset latency	6,8 min.	2,9 min.	22,3 min.	11,2 min.
Sleep latency REM	–	–	161,5 min.	46 min.
Sleep latency N2	147,5 min.	4 min.	1,5 min.	10 min.
AHI	32,6/h	29,4/h	36,8/h	5,8/h
AI	18,9/h	17/h	25,1/h	1,9/h
HI	13,7/h	12,3/h	11,7/h	3,8/h
Obstructive AI	17/h	16,1/h	25/h	1,6/h
Central AI	1,1/h	0,8/h	0/h	0,3/h
Mixed AI	0,9/h	0,2/h	0,2/h	0/h
AHI supine & TST	57,6/h	61,9/h	41,1/h	5,8/h
Apnea maximum	96,6 s.	37,5 s.	62,9 s.	23 s.
Apnea average	19,2 s.	20,4 s.	27,8 s.	14,3 s.
Desaturation index	30,4/h	29,4/h	44,5/h	5,3/h
Average oxygen saturation	92,5%	91,1%	88,6%	94%
Lowest oxygen saturation	82%	85%	74%	84%
Oxygen saturation below 90%	22,4 min.	49 min.	213,2 min.	3,5 min
PLM-Index	26,4/h	1,6/h	0/h	0/h
PLM in wake	53,3%	0%	0/h	0/h
LM in TST	40,5%	9,7%	0,3/h	2,8/h
Arousal in TST	8,1%	3,3%	2%	3,8%

The patient slept with the tongue pacemaker switched off every monitoring night. TIB, time in bed; TST, total sleep time; REM, rapid eye movement sleep stadium; N1, sleep stadium N1; N2, sleep stadium N2; N3, sleep stadium N3; AHI, apnoe-hypopnoe-index; AI, apnoe-index; HI, hypopnoe-index; PLM, periodic limb movement; LM, limb movement.

On the second night, due to the observed augmentation effects, the patient’s medication was changed to Rotigotine 3 mg/24 h and Tilidin 50/4 mg (as necessary). This led to a substantial reduction in the PLM index in the second night and greatly improved sleep efficiency while, as expected, the respiratory parameters remained nearly the same (see Table 1).

Because the patient reported side effects with the Rotigotine-dermal patch, drug treatment with Ropinirole 1 mg (0-0-0-1), retarded Ropinirole 2 mg (0-0-1-0), Tilidin 50/4 mg (as necessary) and administering Pregabalin in successive doses from 25 mg-50 mg-75 mg was prescribed. An effectiveness check was recommended after 3–4 months of this course of treatment.

To check the effectiveness of the drug-based RLS therapy and the sleep apnea syndrome, the patient spent two more nights in our sleep laboratory. During the first control night, the medication showed a marked improvement in periodic leg movements during sleep (see Table 1). As in the previous nights, the tongue pacemaker remained switched off during the nights.

**Figure 2:**
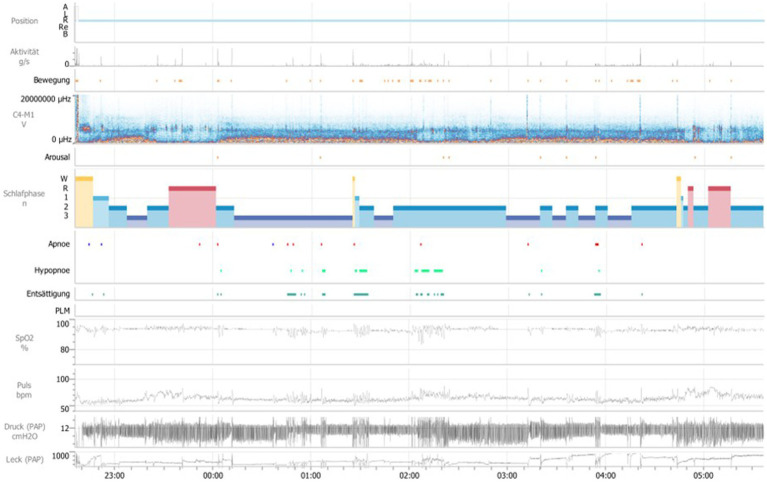
General overview over the second polysomnography, second night. Position A = upright position, Position R = on the back, Position L = left side, Position Re = right side, Position B = on the stomach, Aktivität g/s = activity of limb in sleep, Bewegung = movement, μHZ = microhertz, C4-M1 V = Derivation of EEG electrodes C4 to M1, Schlafphasen W = wake, Schlafphasen R = REM-Sleep, Schlafphasen 1 = sleep stage 1, Schlafphasen 2 = sleep stage 2, Schlafphasen 3 = sleep stage 3, Entsättigung = desaturation, PLM = periodic limb movement, SpO2% = oxygen saturation in percent, Puls bpm = beats per minute, Schnarchintervall = snoring interval, Druck (PAP) cmH2O = pressure (positive airway pressure) in cm water-column, Leck (PAP) = Leakage (positive airway pressure).

Due to significantly improved motor parameters in this first control night, another attempt to initiate APAP-therapy was discussed with the patient. On the second control night, the above-mentioned medication and APAP-therapy (Air Sense 10 Elite, pressure setting of 12 cm H2O) with a full-face-mask and humidifier was applied (Figure 2). Under this therapy, both the sleep-related breathing parameters and the movement parameters have normalized (see Table 1).

## Discussion

This case report presented points out the importance of a combined pneumological and neurological approach to sleep medicine in sleep-specific therapy evaluation. Inadequate therapy for one sleep disorder could have a negative impact on the therapy for another sleep disorder or, as in the case described here, make such therapy appear completely ineffective. For our patient, his RLS also led to excessive periodic movements of the patient’s legs at night, which made APAP-therapy almost impossible.

However, untreated or inadequately treated RLS is not only a severe problem for the patient, but also a major socioeconomic burden for western healthcare systems. Trenkwalder et al. (2) estimate that the socioeconomic impact of restless legs syndrome and inadequate restless legs syndrome management across European settings varies between €20,188.68 million per year, where the prevalence of RLS patients requiring treatment is assumed conservatively to be 1.6% of the population, to €34,068.41 million per year, where the prevalence of RLS patients suffering from moderate to severe RLS is estimated to be 2.7% of the population. Moreover, when considering the overall socioeconomic burden of neurological diseases in an EU subsample, RLS is ranked the fifth largest economic disease burden (with mood disorders as first place, followed by dementia, psychotic and anxiety disorders) and more impactful than stroke, addiction and headache (3).

In our case study, the ineffectively treated RLS with PLMS led initially to rejection of APAP-therapy, and ultimately, to the implantation of a hypoglossal nerve stimulator. There are studies providing evidence that hypoglossal nerve stimulation can improve sleep architecture and decrease arousal index in patients with OSA (4), but according to our patient, this was not the case because he could not detect any significant improvement in his night’s sleep. In addition, although complications from the insertion of the hypoglossal nerve stimulator are rare – mostly from infections – it remains an invasive procedure with all the risks associated with the implantation of a technical device, which may result in lifelong disadvantages for the patient (surgical battery change, limited MRI suitability).

As for the tongue pacemaker commonly used today, another disadvantage of this unit is that the criteria for implanting the tongue pacemaker does not apply to all patients with moderate to severe sleep apnea syndrome, because these patients, in particular, often have comorbid obesity grade II or more and are therefore not suitable candidates for the implantation of the hypoglossal nerve stimulator. For this group of patients, it may be worth efforts to try and improve compliance with nocturnal PAP-therapy.

But compliance alone is often not sufficient for effective PAP-therapy. Effective PAP-therapy is usually defined as a reduction of daytime sleepiness, an improvement in overall daily functioning and recovery of the cognitive functions such as verbal memory (5, 6). In order for PAP-therapy to be effective, an application duration of at least four hours/night for at least five nights/weeks is recommended. Improvements in various aspects of a patient’s health have a positive correlation with the duration of use (5). Impact on the recovery of higher cognitive functioning, for example, has been described in the literature from a PAP-application of 7–8 h per night, but this period of use is unfortunately still an exception (6).

Several studies investigating the factors causing a lack of adherence to this course of treatment have shown that almost 50% of patients discontinue PAP after one year of treatment, while 8 to 15% of patients reject treatment as early as the first night of application (7). Moreover, Rotenberg et al. (7) reviewed 82 studies over a period of 20 years (1994–2015) and found that PAP adherence remained persistently low across all the years, at around 34% (30%–40%). This sustained low rate of adherence is critical and shows that discontinuance of PAP is a global problem (7).

When looking more closely at the reasons for a patient’s lack of adherence, the following factors are often mentioned in the literature: disease severity, possible side effects during the application of the device such as skin irritation, conjunctivitis, nasal congestion, dry throat, and psychological factors such as anxiety disorders/claustrophobia, as well as, socio-demographic/economic characteristics of the patient. Further studies found that in particular, mask air leakage and discomfort have been linked to poor PAP adherence, which led Valentin et al. (8) to speculate that air leakage could be the most promising target for future studies aimed at improving adherence to PAP-therapy.

In our case study, we have shown how much an untreated or insufficiently treated restless legs syndrome can lead to a suboptimal APAP mask fit due to motor restlessness during sleep, and ultimately, to a complete rejection of PAP-therapy. After the successful treatment of the RLS, our patient achieved an uncomplicated application of APAP-therapy. Moreover, the patient expressed greater satisfaction with the APAP-therapy because he felt more rested in the morning, particularly when compared to the tongue pacemaker therapy.

Many studies recommend improving the mask fit to increase compliance for PAP-therapy, but only a few studies looked at the connection between RLS and OSAS and the effects of nocturnal motor restlessness on mask fit although prevalence is high. In a review, Roux et al. (9) found a prevalence of RLS/PLMS and obstructive sleep apnea (OSA) in between 5.5% and 8.3% (10). The correlation between these sleep-related disorders was significantly increased. Interestingly, Jomha et al. (10) explored the prevalence of comorbid sleep disorders in a group of patients with a hypoglossal nerve stimulator (9). They provided evidence for an increased prevalence of insomnia about 47% and restless legs syndrome about 28%. The prevalence for a comorbidity of all three disorders (breathing disorder, insomnia and RLS) was about 14%. These findings of Jomha and colleagues suggest that prevalence of RLS is even higher among patients with hypoglossal nerve stimulator which would support our hypothesis that untreated RLS may be a common reason for APAP therapy failure.

The authors further suspected that obesity may be the linking factor between RLS and OSA because of dysfunctional dopaminergic pathways, which seemed to be crucial in the genesis of RLS, also seemed to be affected in obese people: lower dopamine D2 receptor availability in the striatum of obese people, compared with normal-weight individuals, could contribute to the observed link between RLS and OSA as most OSA patients are obese (10).

Even if the complex mechanisms underlying the association between OSAS, RLS and PLMS remain unclear, this connection is also of crucial importance for adherence to PAP-therapy, shown in our case report, as well as in the additional recording of such comorbidities which ultimately leads us to delineate a more tailored therapy.

## Conclusion

Although long-term studies are still lacking, the method of hypoglossal nerve stimulation appears as a new and encouraging possibility in the treatment of obstructive sleep apnea. But it also requires invasive surgery and the insertion of an electronic device, which – in some cases – might not have been necessary. The observed high correlation between RLS/PLMS and sleep-disordered breathing has some important clinical implications since nocturnal movements induced by PLMS can impair not only sleep continuity, but also PAP-therapy which should be treated accordingly. If we risk a closer look at the causes of PAP intolerance, we can achieve a more effective therapy for our patients.

## Data availability statement

The original contributions presented in the study are included in the article/supplementary material, further inquiries can be directed to the corresponding author.

## Ethics statement

Ethical review and approval was not required for the study on human participants in accordance with the local legislation and institutional requirements. Written informed consent from the patients/participants or patients/participants' legal guardian/next of kin was not required to participate in this study in accordance with the national legislation and the institutional requirements. Written informed consent was obtained from the individual(s) for the publication of any potentially identifiable images or data included in this article.

## Author contributions

CD: Writing – original draft. CB: Funding acquisition, Writing – review & editing.

## Figures and Tables

**Table:** 

AHI	apnea hypopnea index
AI	apnea index
APAP	automatic positive airway pressure
BMI	body mass index
ESS	Epworth sleepiness scale
HI	hypopnea index
LM	limb movement
minSaO2	lowest oxygen saturation
MRI	magnetic resonance imaging
N1	sleep stage N1
N2	sleep stage N2
N3	sleep stage N3
ODI	oxygen desaturation index
OSAS	obstructive sleep apnea syndrome
PAP	positive airway pressure
PLM	periodic limb movement
PLMS	periodic limb movement of sleep
PY	pack years
REM	rapid eye movement
RLS	restless legs syndrome
TIB	total time in bed
TST	total sleep time
